# Address by President Betty

**Published:** 1892-02

**Authors:** 


### THE DENTAL REGISTER.

VOL. XLVI.] FEBRUARY, 1892. [No. 2.

### COMMUNICATIONS.

### Address by President Betty.

PAPER AND DISCUSSION.

The Annual Meeting of the Ohio State Dental Society held in Columbus Dec. 1st to 3rd, inclusive, 1891.

Though I see I have been mentioned on the pragramme for the conventional annual address, I must, nevertheless, this time, honor the custom " more in the breach than in the observance."

It seems clear to me that the presiding officer should exert himself more in wisely and justly administering his executive duties than posing as the reader of the initial paper at such a meeting as this, bringing together as it does the talent from various parts of our own State and distinguished visitors from others, all of whom have come prepared to present matter of much importance. It is gratifying to note that so many have taken a hand to keep the cause of education in its forward march, and I sincerely believe that the present meeting will add much to the fund from which all draw their supplies. Every mite that is added is so much gain to that training, that experience which we denominate " education," and every one should feel proud to be a contributor.

I should like to express my approval of the new interest this society has taken in clinics, and that the beginning made this year will be but an earnest effort of what we may expect in the future, and to that end we can, one and all, assist very materially if we only determine so to do.

Although great advance has been made within the past several years both in scientific and applied dentistry, as well as in college training, and the increased number and quality of our periodicals, we have yet much to learn and much to do, and as the wish is father to the thought, we will now proceed to business.

